# Evaluation of the Antioxidant, Anti-Inflammatory and Cytoprotective Activities of Halophyte Extracts against Mycotoxin Intoxication

**DOI:** 10.3390/toxins13050312

**Published:** 2021-04-27

**Authors:** Nolwenn Hymery, Xavier Dauvergne, Halima Boussaden, Stéphane Cérantola, Dorothée Faugère, Christian Magné

**Affiliations:** 1 Laboratoire Universitaire de Biodiversité et Écologie Microbienne, Université de Brest, EA 3882, F-29280 Plouzané, France; dorothee.faugere@orange.fr; 2 Géoarchitecture Territoires, Urbanisation, Biodiversité, Environnement, Université de Brest, EA 7462, CS 93837, CEDEX 3, F-29238 Brest, France; xavier.dauvergne@univ-brest.fr (X.D.); hboussaden@gmail.com (H.B.); Christian.Magne@univ-brest.fr (C.M.); 3 Service Général des Plate-Formes Technologiques, Plateforme RMN-RPE, Université de Bretagne Occidentale, CS 93837, CEDEX 3, F-29238 Brest, France; stephane.cerantola@univ-brest.fr

**Keywords:** animal cell lines, antioxidant activity, cytotoxicity, halophytes, in vitro, mycotoxins

## Abstract

Twelve halophyte species belonging to different families, widely represented along French Atlantic shoreline and commonly used in traditional medicine, were screened for protective activities against mycotoxins, in order to set out new promising sources of natural ingredients for feed applications. Selected halophytic species from diverse natural habitats were examined for their in vitro anti-mycotoxin activities, through viability evaluation of Madin-Darby Bovine Kidney (MDBK) and intestinal porcine enterocyte (IPEC-J2) cell lines. Besides, the in vitro antioxidant activities of plant extracts were assessed (total antioxidant and 2,2-diphenyl-1-picrylhydrazyl (DPPH)-scavenging bioassays). Of the 12 species, *Galium arenarium*, *Convolvulus soldanella* and *Eryngium campestre* exhibited the most protective action on MDBK and IPEC-J2 cells against zearalenone (ZEN) or T2 toxin contamination (restoring about 75% of cell viability at 10 μg·mL^−1^) without inflammation response. They also had strong antioxidant capacities (Inhibitory concentration of 50% (IC_50_) < 100 μg·mL^−1^ for DPPH radical and total antioxidant capacity (TAC) of 100 to 200 mg Ascorbic Acid Equivalent (AAE)·g^−1^ Dry Weight), suggesting that cell protection against intoxication involves antioxidant action. A bio-guided study showed that fractions of *G. arenarium* extract protect MDBK cells against T2 or ZEN toxicity and several major compounds like chlorogenic acid and asperuloside could be involved in this protective effect. Overall, our results show that the halophytes *G. arenarium*, *C. soldanella* and *E. campestre* should be considered further as new sources of ingredients for livestock feed with protective action against mycotoxin intoxication.

## 1. Introduction

Cereal grains, as critical crop products, play a crucial role in the human diet and livestock feed due to their valuable contents such as carbohydrates, proteins, fatty acids and vitamins [1]. The feed supply chain is a crucial element for all livestock production systems. According to the FEFAC [2], within the European Union-28 (EU-28), approximately 475 million tons of feedstuffs and forages are consumed by livestock each year. For example, France ranks the 1st among European countries in the production of durum wheat.

About one fourth of global agricultural products and half of cereal grain samples (or derived products) used for livestock feed are contaminated with mycotoxins in Europe and worldwide [3,4,5]. Fumonisins, trichothecenes and zearalenone (ZEN) are the most commonly occurring fusariotoxins in cereal grains and animal feed [6,7]. Regarding finished feed, FBs (91%), deoxynivalenol (82%), ZEN (35%), aflatoxins (18%), T2 (4%) and ochratoxin A (3%) are the most frequently found mycotoxins under warm climate conditions [8,9]. Both ZEN and deoxynivalenol (DON) are produced in the field and upon postharvest storage (grain, silage), whereas T2 is produced only upon seed storage. All 3 are toxic to livestock, particularly monogastric animals which do not possess rumen with mycotoxin-degrading microorganisms [10]. ZEN interacts with estrogen receptors, inducing reproduction troubles (abortion, impairment of fertility etc.) [11,12]. T2 inhibits protein and DNA synthesis, and causes emaciation, diarrhea, immunosuppression, skin necrosis and haemorrhage [13,14]. DON decreases weight gain and vaccination response, and causes vomiting and rejection of feed [10,15,16].

Despite efforts to control fungal contamination, including the implementation of good agricultural and manufacturing practices, extensive mycotoxin contamination has been reported to occur in feed and food. At the field level, crop rotation, variety selection, irrigation, tillage, fungicide spray may allow a reduction of toxin-producing fungi or of mycotoxin prevalence. However, the presence of fungi or toxins within cereal products still remains a problem.

Upon storage of cereal products, addition of adsorbants such as clays (e.g., Mycofix^®^, Biomin or Amadeite^®^, Olmix, Brehan, France), active charcoal or seaweed-extracted biopolymers may reduce toxin bioavailability. Besides, a number of chemical or biological treatments have been proposed to transform or scavenge mycotoxins [17]. They include ammoniation or gamma irradiation for seed decontamination but are rather inefficient against fusariotoxins or inapplicable by breeders [18]. Moreover, the use of probiotics like micro-organisms and derived products as detoxifying agents [19] has been proposed. However, these probiotics hardly affect some mycotoxins like DON [20]. Moreover, most of the results are highly variable depending on the method and the mycotoxin used. Therefore, an efficient natural food solution protecting livestock against deleterious effects of mycotoxins is still needed.

Plants have long been a source of medicines and phytoproducts are widely used in the food industry, nutraceuticals, and medicine. The latest trend of returning to natural sources for health has generated a wide interest for bioactive compounds from plants. For instance, the antioxidant plants have gained a huge amount of attention during the last two decades and the supplementation of exogenous antioxidants appears as a promising way to improve the undesirable effects of oxidative stress. In that context, halophytes represent a renewable source of bioactive molecules for food, cosmetics, and pharmaceutical uses. These plants have been poorly studied until the last decade, and recent works showed that they exhibit numerous biological effects thanks to their high levels of secondary metabolites [21,22]. Among them, polyphenols display significant antioxidant activities and a wide spectrum of medicinal properties [23,24,25].

At a cellular level, one of the main effects induced by mycotoxins on consumers is a significant oxidative stress and inflammatory process [26]. Since halophytic plants have a constitutively strong antioxidant defense, we investigated the potent protective effect of edible halophytes against mycotoxin contamination in animal cells. Such property was studied along with the antioxidant and anti-inflammatory potential of these plants to assess the possible mechanisms of this cytoprotective action.

## 2. Results

### 2.1. Antioxidant Activities of Halophytes

**Figure 1:**
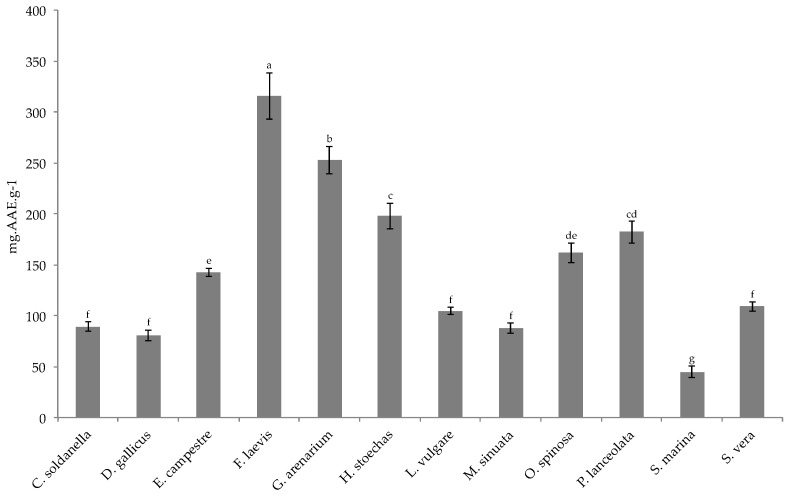
Total antioxidant capacity (mg Ascorbic Acid Equivalent (AAE)·g^−1^ DW) of the 12 halophytic crude extracts. Means ± SD of three replicates are represented, and different letters above the bars indicate significantly different means (*p* < 0.05).

The twelve studied species differed in their total antioxidant capacity (Figure 1). Of these species, *F. laevis* and *G. arenarium* exhibited the highest activity with total antioxidant capacity (TAC) values of 315.6 and 251.8 mg Ascorbic Acid Equivalent (AAE)·g^−1^ Dry Weight (DW), respectively. Conversely, *C. soldanella, D. gallicus*, *L. vulgare*, *M. sinuata*, *S. marina*, and *S. vera* extracts showed a moderate antioxidant capacity, with less than 100 mg AAE·g^−1^ DW.

**Figure 2:**
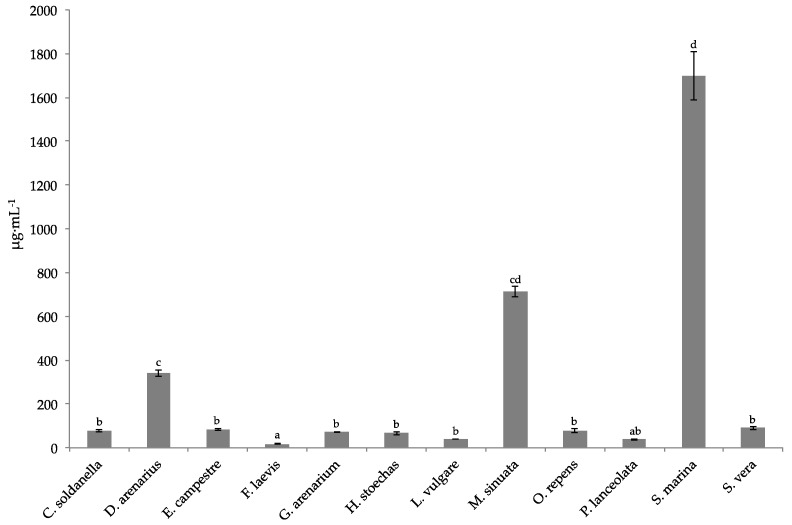
Radical scavenging activity against 2,2-diphenyl-1-picrylhydrazyl (DPPH) (μg·mL^−1^) of the 12 halophytic crude extracts. Means ± SD of three replicates are represented, and different letters above the bars indicate significantly different means (*p* < 0.05).

The same trend was obtained with the 2,2-diphenyl-1-picrylhydrazyl (DPPH) assay, where *F. laevis* exhibited the highest activity with Inhibitory concentration of 50% (IC_50_) of 18 μg·mL^−1^, followed by *P. lanceolata* (Figure 2). Overall, all the halophytes showed a strong antiradical activity (IC_50_ < 100 μg·mL^−1^) except *S. marina*, *D. gallicus* and *M. sinuata*.

### 2.2. Cytotoxicity of Mycotoxins and Animal Cell Protection by Halophyte Extracts

#### 2.2.1. MDBK Cells

Preliminary studies showed no cytotoxicity of plant extracts at concentrations up to 10 µg·mL^−1^ (data not shown). Conversely, mycotoxins at 10 µM caused a 40% to 50% decrease of cell viability. Cells co-cultured with each plant extract and T2 or ZEN exhibited less mortality than cells incubated with ZEN or T2 alone (Table 1). Concerning cytotoxicity of DON, no statistical differences were observed for cells cultured with or without plant extracts (60% of viability). *G. arenarium* extract induced the highest viability restoration (74.4%) of cells exposed to T2, followed by *C. soldanella* (74.0%). About 77.2% of Madin-Darby Bovine Kidney (MDBK) cells exposed to ZEN and preincubated with *G. arenarium* extract were viable, vs. only 62% without *G. arenarium* extract. The extracts from *E. campestre* (76.8%), *L. vulgare* (76.5%) and *O. spinosa* (76.1%) were also efficient in restoring cell viability.

In order to study anti-inflammatory effects of plant extracts, cells were cultured with halophyte extracts and mycotoxin, and proinflammatory factors were measured. Each plant extract alone did not statistically affect TNF-α and IL-8 productions by MDBK cells (data not shown). In the presence of halophytes extracts, TNF-α and IL-8 secretions are similar to controls (Table 2).

When cells are exposed to DON and T2 in the presence of halophytes, TNF-α and IL-8 productions decreased compared to cells treated with DON or T2 alone. Exposure to ZEN was no effect on TNF-α and IL-8 productions. Plant extracts had no effect on proinflammatory activity (data not shown).

#### 2.2.2. IPEC-J2 Cells

The extracts showing the strongest effect on MDBK cells were selected to be tested on intestinal porcine enterocyte (IPEC-J2), namely *C. soldanella*, *E. campestre*, *G. arenarium*, *L. vulgare* and *O. spinosa*. In the presence of plant extracts, cell viability was not affected and remained close to 100% (data not shown). Conversely, exposure of IPEC-J2 cells to T2, ZEN or DON toxin drastically decreased viability (to 52%, 62% and 60%, respectively) (Table 3). Preincubation of the porcine cells with each plant extract significantly restored viability upon T2 or ZEN treatment, but had no effect against DON intoxication. A total recovery of cell viability was found in the presence of *E. campestre* extract upon T2 exposure, followed by *C. soldanella* and *G. arenarium*, whereas the latter was the most efficient extract upon ZEN intoxication, followed by *E. campestre* and *O. spinosa*.

Concerning TNF-α or IL-8 production, as MDBK cells, a decrease has observed in the presence of plant extracts compared to cells incubated with DON and T2 alone. ZEN was not effect on proinflammtory factors (Table 2). Plant extracts were no effect on proinflammatory activity (data not shown).

This step led us to select *Galium arenarium* extract, exhibiting strong antioxidant and cytoprotective effects, for a bioguided study aimed at identifying and characterizing valuable fractions and compounds.

### 2.3. Bioguided Purification of Gallium arenarium Extract and Cytoprotective Effect on MDBK Cells

#### 2.3.1. Characterization of *G. arenarium* Fractions

**Figure 3:**
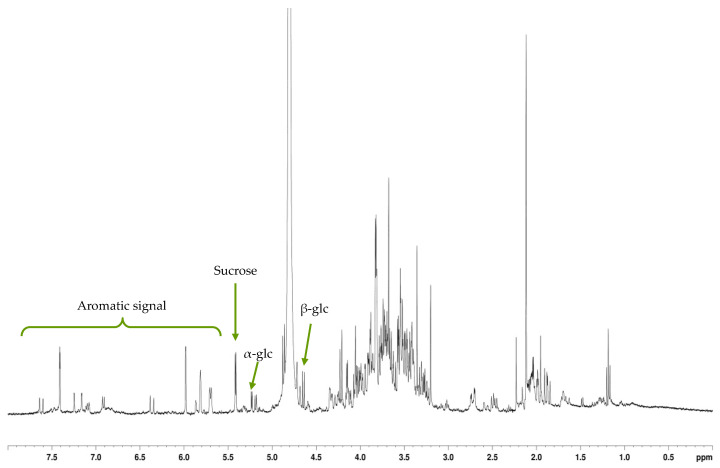
^1^H-NMR spectrum of *G. arenarium* raw extract.

**Figure 4:**
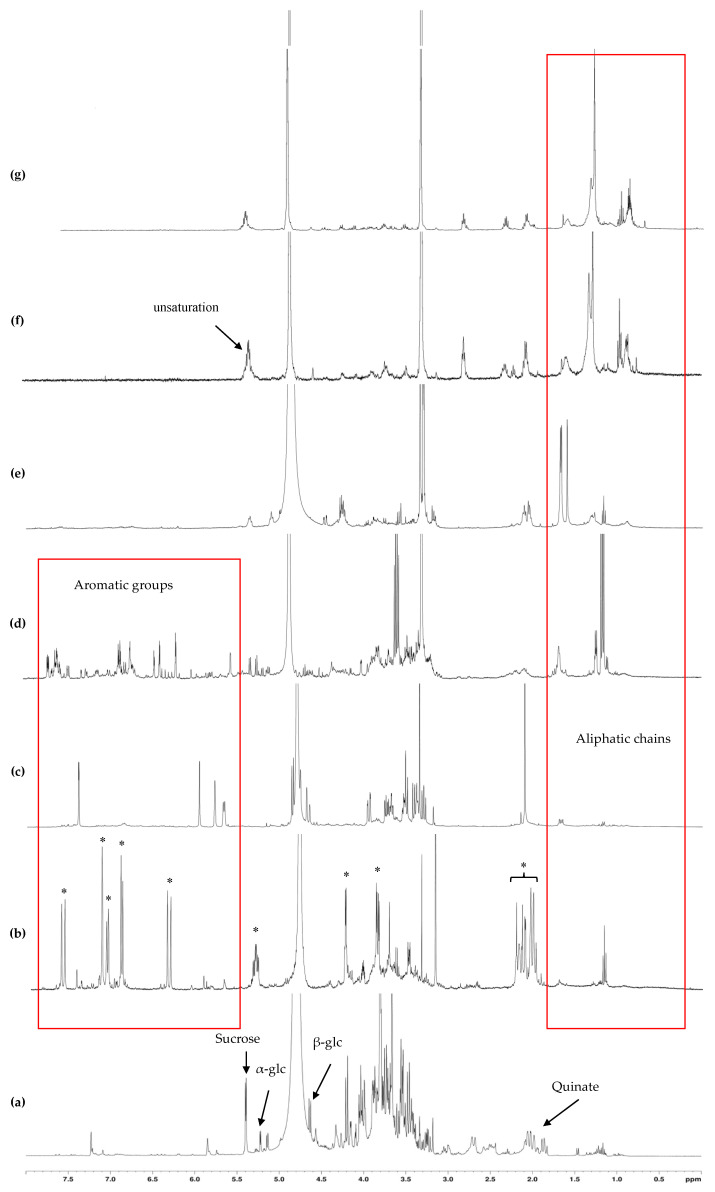
^1^H-NMR spectra of *G. arenarium* fractions. (**a**) water; (**b**) 20% methanol; (**c**) 40% methanol; (**d**) 80% methanol; (**e**) 100% methanol; (**f**) ethanol; (**g**), ethyl acetate. *, characteristic signals of chlorogenic acid.

Characterization of *G. arenarium* extract and its fractions was performed by nuclear magnetic resonance (NMR) spectroscopy. Accordingly, the ^1^H-NMR spectrum of the raw extract showed the presence of sucrose, glucose, and a number of signals in the aromatic and aliphatic zones (5.5 to 7.5 ppm and 1 to 2.5 ppm, respectively) (Figure 3). Carbohydrates were eluted in the water fraction (effluent, Figure 4a). MeOH_20_ fraction showed mainly signals of bound forms of quinate (multiplet at 1.8 ppm), indicating the presence of chlorogenic acid (Figure 4b). NMR spectrum of the MeOH_40_ fraction showed characteristic signals, including a doublet at 5.7 ppm and singlets at 5.8, 5.95 and 7.4 ppm (Figure 4c). Further purification of this fraction by HPLC allowed us to obtain a pure compound, which provided glucose, a terpenoid and a lactone following acid hydrolysis. Finally, ^13^C- and 2D NMR experiments led us to identify the iridoid glycoside asperuloside (Table 4). Mass spectrometry analysis unequivocally confirmed this characterization, with molecular ion of *m*/*z*: 436.929 (theoretical *m*/*z*: 437.101 [M + Na]).

The complex ^1^H-NMR spectrum of the MeOH_60_ fraction exhibited several signals between 6 and 7.6 ppm which mark the presence of several aromatic compounds with different bonding (Figure 4d). The MeOH_80_ fraction showed several multiplets in the anomeric region and more pronounced signals between 1.5–1.8 ppm on its ^1^H-NMR spectrum, corresponding to aliphatic protons (Figure 4e). The last two fractions, eluted with MeOH_100_ and ethyl acetate, exhibited similar apolar compounds with aliphatic protons, as shown by the intense signals in the 0.8–1.4 ppm region (Figure 4f,g). Some signals at 5.2 ppm, characterizing unsaturations, suggest the presence of terpenoids in these two apolar fractions.

#### 2.3.2. Effects of Mycotoxins and *Galium arenarium* Extracts on the MDBK Cell Membrane Integrity

Trans-epithelial electrical resistance (TEER) was assessed in MDBK cells upon mycotoxin exposure (Table 5). In the presence of *Galium arenarium* extract or EtOH fraction alone, TEER was not affected. However, exposure to T2 and ZEN dramatically decreased TEER to 66 and 73% of the control, respectively. Cells exposed to T2 in the presence of *G. arenarium* extract or its EtOH fraction showed a significant TEER recovery, increasing to 88% and 87% of the control, respectively. Upon exposure to ZEN, TEER increased to 92% of the control in MDBK preincubated with *G. arenarium* extract or its EtOH fraction.

Considering mitochondrial activity, evaluated with MTS assay, all fractions tested showed the same effect. These fractions were more efficient than crude extract in maintaining MDBK cell viability in the presence of DON (Table 6). They were as powerful as the crude extract and partially protected cells of T2 and ZEN intoxication.

## 3. Discussion

Mycotoxins and their control generate a considerable economic impact not only to the feed producer but also to intermediaries such as elevators, buyers of grains and food processors. Previous studies have described the toxicological effects of *Fusarium* toxins in farm animals [27,28]. In ruminants, the animal species least sensitive to DON, feed refusal syndrome was reported in cows after consumption of wheat contaminated with 6.4 mg DON/kg feed during 10 weeks [29]. In pigs, the symptoms include vomiting, diarrhea, leukopenia, hemorrhage, shock and death. Toxic effects of T2 toxin are usually manifested in the form of Alimentary Toxic Aleukia (ATA). Ruminants are known to be relatively resistant to T2 toxin in comparison to monogastric animals. The toxic effects of ZEN on cows are associated with vulvar hypertrophy and ovarian atrophy. Ruminants are less susceptible than pigs to this toxin due to ruminal microbiota’s potential to transform ZEN to its hydroxyl metabolites, α-zearalenol and β-zearalenol. Some solutions have been described as addition of various inorganic ligands such as aluminosilicates, hydrated sodium calcium aluminosilicate and zeolite prevented the adverse effects of aflatoxins on swine performance. However, this anti-mycotoxin ability of these products could not prevent the absorption of *Fusarium* toxins in the intestine of pigs [30]. Than et al. [31] determined the adverse effects of four commercial anti-mycotoxin additives in preventing the negative effects of DON. Anti-mycotoxin additives were ineffective at improving the growth performance at dietary concentrations used.

Over the past decades, the search for natural products in plants has led to the discovery of a number of biologically active substances, particularly secondary metabolites, which confer plants with a number of biological activities. Of the twelve species studied here, nine exhibited very strong antioxidant capacities (IC_50_ < 100 μg·mL^−1^ for DPPH radical and TAC higher than 100 mgAAEg-1DW), reinforcing the idea that halophytes are constitutively equipped with an efficient antioxidant system to cope with harmful coastal environments [21,32]. Moreover, it has already reported that antioxidant compounds may alleviate mycotoxin toxicity [33]. Here, the studied halophytes showed a strong protective action on MDBK and IPEC-J2 cells against ZEN or T2 toxin contamination (restoring about 75% of cell viability at 10 μg·mL^−1^). However, ranking of antioxidant activities of the studied species did not fully match with that of cytoprotective action upon mycotoxin exposure, suggesting that cell protection against intoxication would require additional mechanisms along with antioxidant action. In particular, every extract failed to protect animal cells against DON intoxication though they all showed antioxidant effects. The main toxic mechanism of DON is well-known. DON binds to ribosomes thereby destroying their structure. It is also reported to interfere with the peptidyl transferase active center existing on the 60S subunit of ribosomes thus inhibiting normal protein synthesis [15]. At the molecular level, DON induces the global impact on the transcriptome and triggers prototypical signaling pathways linked to immunity and inflammation, including the p38 mitogen-activated protein kinases (p38-MAPK) and the NF-κB [31]. Same effects were described on IPEC-J2, DON exposure alters the phosphorylation states and sites of multiple proteins found in differentiated intestinal epithelial cells [34]. On MDBK, exposure to DON suggested decreased protein synthesis [35]. Further investigations are necessary to analyze this intriguing mechanism of DON in the intestine and renal cell models.

This preliminary study allowed us to select the most efficient extract in terms of antioxidant capacity and cytoprotective effects, namely that of *Galium arenarium*, for further analyses addressed to identify the potent bioactive compounds. Moreover, such bioguided study was performed on MDBK cells, a relevant model to study bovin nephrotoxicity of mycotoxin and since it possessed a shorter generation time.

Several studies have investigated chemical composition and assessed biological properties of crude extracts from *Rubiaceae* [36,37]. However, no studies so far have investigated thoroughly the molecules responsible for these activities. Here, enriched fractions of *G. arenarium* extract have been evaluated for the first time for antioxidant, anti-inflammatory and cytoprotective activities, particularly in the context of mycotoxin exposure.

The crude extract of *G. arenarium* exhibited a strong antioxidant activity, confirming previous works with other bioassays [38]. The bio-guided fractionation of this extract allowed us to recover and concentrate the antioxidant activity into four fractions, eluted with 20 to 80% methanol. Accordingly, these fractions exhibited a DPPH IC_50_ lower than 40 µg·mL^−1^ and total antioxidant capacity higher than 300 mg AAE·g^−1^ DW. Interestingly, these fractions all exhibited a strong protective action on bovine and porcine cells upon T2 or zearalenone exposure, suggesting that this effect is at least due to the relevant phenolic compounds present in these fractions, as it has been already reported [39,40,41]. Accordingly, Montibus et al. [42] showed that the phenolic composition of a maritime pine extract, and particularly methylated compounds, would be essential for its antifungal and anti-mycotoxin properties.

The fraction eluted with 20% MeOH exhibited a high phenolic content and, correlatively, a strong antioxidant activity. NMR analyses of this fraction showed that its major constituents were free quinic acid and its bound form chlorogenic acid, a well-known antioxidant compound already reported in *Galium* genera [43,44].

The fraction eluted with 40% MeOH exhibited the strongest antioxidant capacity. 2D-NMR analyses of this fraction showed the predominance of the iridoid glycoside asperuloside. Such compound has already been reported in some *Galium* species [37,45] but never in *G. arenarium*. It has been related to a number of pharmacological properties as an anticancer [46], anti-inflammatory and anti-obesity [47], anti-viral, anti-malarial, anti-protozoal, anti-hypertensive, immunomodulatory, and antioxidant agent [48]. Therefore, we suggest that the abundance of this iridoid is responsible for the very strong antioxidant and anti-inflammatory activities of the MeOH_40_ fraction. Moreover, it could be involved in the cytoprotective action of *G. arenarium* MeOH_40_ fraction.

The fraction eluted with 60% MeOH exhibited the strongest DPPH-scavenging activity (Table 7). The major compounds characterized in this fraction were glycosylated phenolic compounds. Considering the strong antioxidant power of phenolic glycosides [49], these compounds are likely to be responsible for the strong antioxidant activity of this fraction. Moreover, the strong anti-inflammatory and cytoprotective actions of MeOH_60_ fraction could be due to such phenolic glycosides, as it has been already reported [34]. Besides, these activities could also be attributed to anthraquinones, as reported in several *Galium* species [50,51].

The fraction eluted with 80% MeOH exhibited no signals of aromatic compounds but still higher antioxidant activities than those of the crude extract. Noteworthily, this fraction exhibited a remarkable cytoprotective activity. Along with the 1D- and 2D- NMR analyses, such activities could be due to terpenoids as reported by Grassman [52].

The last two fractions showed low antioxidant activities but still a strong cytoprotective action against mycotoxin exposure (particularly T2 toxin and zearalenone), indicating that cytoprotective action against mycotoxin intoxication does not only proceed through antioxidant pathways. It is supposed here that another mechanism of cell protection like anti-inflammatory process would also provide cell protection effect. Moreover, their NMR spectra showed several strong signals in the aliphatic region, which could be attributed to phytosterols such as sitosterol or campesterol, as already reported [53].

## 4. Conclusions

Of the twelve halophytic species studied here, several exhibited substantial protective effects on animal cells against mycotoxin. Moreover, these species have strong antioxidant activities. Further experiments are under progress to identify the different molecules responsible for this anti-mycotoxin activity, particularly in the less polar fractions. Besides, it will be crucial to evaluate potent curative effects and extend this work to in vivo experiments on livestock (bovine, porcine, chicken or fish), before these extracts could be proposed as a new source of feed ingredients.

## 5. Materials and Methods

### 5.1. Chemicals, Culture Media and Supplements

Madin-Darby Bovine Kidney (MDBK) and intestinal porcine enterocyte (IPEC-J2) cell lines were purchased from DSMZ, (Braunschweig, Germany). Cell culture medium (DMEM, EMEM, Eagle’s Minimum Essential Medium), horse fœtal serum (HSF), Folin-Ciocalteu phenol reagent, DPPH, all standards and solvents used for chemical analyses were supplied by Sigma Aldrich (St. Louis, MO, USA). Concerning mycotoxins, deoxynivalenol (DON) (MW = 296.35 g/mol, purity ≥ 98%) is produced by *Fusarium* sp. T2 (MW = 466.5 g/mol, purity ≥ 98%) and zearalenone (ZEN) (MW = 320.38 g/mol, purity ≥ 98%) have a not specified origin. All mycotoxins were purchased from Sigma Aldrich.

### 5.2. Cell Culture

MDBK and IPEC-J2 cells were maintened at 37 °C under 5% CO_2_ and water saturated atmosphere. IPEC-J2 is a non-transformed, permanent intestinal cell line. The IPEC-J2 cells were cultured in Dulbecco’s Modified Eagle Medium (DMEM) and supplemented with 1% HEPES, 10% fetal bovine serum (FBS), 1% penicillin/streptomycin. MDBK were cultured in medium (EMEM) containing 4.5 g/L glucose for cell growth was added with 10% horse fœtal serum (HSF) and 1% penicillin/streptomycin to support cell division.

### 5.3. Plant Sampling

Aerial parts of twelve halophytic species were collected along the Brittany shoreline of Finistère (France): *Convolvulus soldanella* L. (Convolvulaceae), *Dianthus gallicus* Pers. (Caryophyllaceae), *Eryngium campestre* L. (Apiaceae), *Frankenia laevis* L. (Frankeniaceae), *Galium arenarium* Loisel. (Rubiaceae), *Helichrysum stoechas* (L.) Moench (Asteraceae), *Limonium vulgare* Mill. (Plumbaginaceae), *Matthiola sinuata* (L.) R.Br. (Brassicaceae), *Ononis spinosa* subsp. *procurrens* Wallr. (Rosaceae), *Plantago lanceolata* (L.) (Plantaginaceae), *Spergularia marina* (L.) (Caryophyllaceae), and *Suaeda vera* Forssk. ex J.F.Gmel. (Amaranthaceae). These species belong to eleven different families (Table 8) and grow in three different habitats (cliffs, sand dune, salt marsh). One sample of each species was deposited at the herbarium of University of Brest. The aerial parts of every sample were rinsed and the leaves were frozen and subsequently freeze-dried. Each sample was then ground to a fine powder in an AE 200 blender (Mettler, Viroflay, France), before extraction and analyses.

### 5.4. Extraction of Metabolites from Halophyte Leaves

About 500 mg of dry powder were homogenized with 5 mL water/ethanol (1:2) under magnetic stirring at 4 °C for 20 min. After centrifugation of the mixture (15 min at 4 °C, 4000× *g*), the resulting pellet was extracted twice following the same protocol. The supernatants were collected, pooled and filtered over glass wool. The obtained extract was concentrated by rotary evaporation at 40 °C and resuspended in either DMSO (for cell treatments) or 50% ethanol (for chemical analyses).

### 5.5. Evaluation of Plant Extract Effects on Cell Lines

#### 5.5.1. Cell Treatments

Cells (MDBK and IPEC-J2) were seeded at 5.10^5^ cells/mL in 96 well plates. After one day of culture, plant extracts were added (0.1–10 μg·mL^−1^). Twenty-four hours later, cells were incubated with cytotoxic concentration of mycotoxins (DON, ZEN or T2) during 48 h before assay analysis.

#### 5.5.2. Measurement of the Epithelial Barrier Function

Measurement of the trans-epithelial electrical resistance (TEER) was performed to evaluate monolayer integrity and possible damage of the cellular monolayer during the experiments. TEER was measured under sterile conditions using the Millicell ERS system (Millipore Co., Bedford, MA, USA) according to the manufacturer’s instructions. TEER values were recorded every 24 h and expressed as Ω × cm^2^ on the basis of the following equation: TEER = (R − Rb) × A, where R is the resistance of filter insert with cells, Rb is the resistance of the filter alone and A is the growth area of the filter in cm^2^.

#### 5.5.3. Evaluation of Cell Viability

The cytotoxic effect of polar and apolar extracts was studied on MDBK and IPEC-J2 cells. Thus, cytotoxicity was evaluated using the CellTiter 96AQueous One cell proliferation assay (Promega, Madison, WI, USA), as described by Hymery et al. [54] (2014). This colorimetric method determines cell viability based on the reduction of 3-(4,5-dimethylthiazol-2-yl)-5-(3-carboxymethoxyphenyl)-2-(4-sulfophenyl)-2H-tetrazolium (MTS) to formazan by mitochondrial dehydrogenases in viable cells. After time of incubation in the presence of samples, cells were washed with PBS, resuspended in 100 μL of the same buffer, and seeded in 96-well plates at 37 °C, under 5% CO_2_ atmosphere and 100% humidity. Then, 20 μL of CellTiter 96AQueous One solution were added to each well and the cells were further incubated for 3 h at 37 °C, under 5% CO_2_ atmosphere and 100% humidity. The absorbance was measured at 490 nm. Cytotoxicity was expressed as the concentration of samples inhibiting cell growth compared with the control (cells treated with 1% DMSO). All tests and analyses were run in triplicate and averaged.

#### 5.5.4. Measurements of Cytokine and Interleukin Productions

MDBK and IPEC-J2 cell lines were incubated for 24 h in the presence of plant extracts. To evaluate a possible antiinflammatory effect of plant extract, IL-8 and TNF-α were monitored. The amount of Tumor Necrosis Factor alpha (TNF-α) and IL-8 in the supernatants of plant extract treated cell cultures was then quantified using ELISA kits (Promocell, Heidelberg, Germany). Three plant extracts were chosen from cytotoxicity results.

### 5.6. Measurement of Antioxidant Activities in Plant Extracts

#### 5.6.1. Total antioxidant Capacity (TAC)

Total antioxidant capacity of ethanolic extracts was evaluated through the assay of a green phosphate/Mo5+ complex according to the method described by Prieto et al. [55]. An aliquot (0.1 mL) of diluted samples was combined with 1 mL of reagent solution (0.3 N sulfuric acid, 28 mM sodium phosphate and 4 mM ammonium molybdate). Methanol was used instead of the sample for the blank. The tubes were incubated in a boiling water bath for 90 min. Then, the samples were cooled to room temperature and the absorbance was measured at 695 nm against blank in UV-Visible spectrophotometer (Anthelie Advanced 2, Secomam, Champigny sur Marne, France). Antioxidant capacity was expressed as mg ascorbic acid equivalent per gram dry weight (mg AAE g^−1^ DW). All samples were analyzed in triplicate.

#### 5.6.2. DPPH Scavenging Activity

The scavenging activity of the stable 1,1-diphenyl-2-picrylhydrazyl (DPPH) free radical was determined by the method of Marwah et al. [56]. Briefly, the reaction medium contained 100 μL of 100 μM DPPH violet solution in ethanol and 100 μL of plant extracts at different concentrations (or water for the control). The reaction mixture was incubated in the dark for 15 min and the absorbance was recorded at 517 nm on a microtiter reader (Multiskan EAR 400, Labsystems, Thermo Scientific, Madison, WI, USA). The assay was carried out in triplicate and butylated hydroxytoluene (BHT) was used as a positive control. The decrease in absorbance upon addition of test samples was used to calculate the inhibition percentage (%IP) of DPPH radical, following the equation:

%IP = [(A_c_ − A_s_)/A_c_] × 100 (1)

where A_c_ and A_s_ are the absorbances of the control and the test sample, respectively. From a plot of concentration against %IP, a linear regression analysis was performed to determine the antiradical activity, as expressed by the IC_50_ (extract concentration resulting in a 50% inhibition) value for each sample.

### 5.7. Fractionation of Plant Extracts

Fractionation of *Galium arenarium* raw extract was performed by solid-liquid partition chromatography on C18-bound silica gel. The elution of polar compounds was made with increasing methanol concentrations (successively 0, 20, 40, 60, 80, 100%) and finally by ethanol and then by ethyl acetate. The fractions were then concentrated by rotary evaporation at 40 °C and resuspended in the corresponding solvent.

### 5.8. Solute Purification

When necessary, purification from one fraction was performed by HPLC using a Shimadzu UFLC XR device equipped with a PDA detector (SPD-M20A, Shimadzu, Kyoto, Japan). A Spherisorb ODS2 column (5 μm, 250 × 4.6 mm, Waters, Milford, MA) was used for solute separation, and the mobile phase consisted of a mixture of acetonitrile 100% (A) and ultrapure water (B). The following linear gradient was applied: t = 0 min 100% B; t = 10 min 100% A; t = 12 min 100% A; t = 15 min 100% B. Compounds were detected at 254 nm and collected. When needed, some of them were submitted to acid hydrolysis treatment (1 N HCl, 110 °C for 1 h) before structural elucidation.

### 5.9. NMR Analyses

For bioactive compound characterization, an aliquot of *G. arenarium* crude extract and each fraction was concentrated by rotary evaporation at 35 °C, and the dry residue was solubilized in deuterated-water (D_2_O) or methanol (MeOD) for NMR analyses. ^1^H- NMR spectra were obtained using a Brüker Avance DRX-400 spectrometer (400 MHz), equipped with a 5 mm TBI probe (^1^H, X, ^31^P) with z gradient (Brüker, Rheinstetten, Germany). A typical 1D ^1^H-NMR spectrum consisted of 32 scans. The determination of major solutes present in sea fennel extract or fractions was made on NMR spectra in comparison with external standards. All ^13^C (J-mod) and 2D Homo- and heteronuclear NMR analyses (COSY, HMBC, HMQC experiments) were performed on a Brüker Avance III HD500 spectrometer equipped with a 5 mm TCI cryoprobe (^1^H, ^13^C, ^15^N) with z gradient. Data analysis was performed using TopSpin^®^ software, 4.0 (Brüker).

### 5.10. Mass Spectrometry Analyses

Mass spectrometry analysis was performed using an Autoflex III smartbean spectrometer (Bruker Daltonics, Billerica, MA, USA). Datas were acquired with Flexcontrol software and analyzed with FlexAnalysis software. The experiments were carried out in positive reflectron mode over a mass range of 0 to 1500 *m*/*z*. The mixture (1 μL of product with 1 μL of matrix) was deposited on a metal plate. The matrix used is of the 4-hydroxy-α-cinnamic Acid (HCCA) type at 10 mg·mL^−1^ in 60% acetonitrile and 40% 0.1% TFA.

### 5.11. Statistical Analyses

All extractions and assays were conducted in triplicate. Results were expressed as mean ± standard deviation (SD), and the means were compared by using one-way analysis of variance (ANOVA) followed by Duncan’s multiple range tests performed by the ‘Statistica v. 5.1′ software (Statsoft, Tulsa, OK, USA). Differences between individual means were deemed to be significant at *p* < 0.05. For antioxidant bioassays, the IC_50_ values were calculated by the sigmoidal fitting of the data using the GraphPad Prism v. 5.0 program (GraphPad Software, San Diego, CA, USA).

## Figures and Tables

**Table 1:** Anti-mycotoxin activity on cell viability of the twelve halophyte extracts, expressed in % of Madin-Darby Bovine Kidney (MDBK) cell viability. Bovine cells were pre-cultivated in the presence of plant extract (10 μg·mL^−1^), then with each mycotoxin (10 μM). Means ± SD of three replicates are represented, and * indicates significantly different means from control cells incubated with mycotoxin alone (*, *p* < 0.05; **, *p* < 0.01).

Halophytic Species	T2	ZEN	DON
*Convolvulus soldanella*	74.0 ± 1.2 **	75.3 ± 0.7 *	61.1 ± 1.3
*Dianthus gallicus*	71.8 ± 0.2 *	72.9 ± 2.6 *	60.4 ± 1.0
*Eryngium campestre*	73.8 ± 0.8 *	76.8 ± 2.0 **	61.8 ± 1.6
*Frankenia laevis*	70.4 ± 4.0 *	72.8 ± 1.2 *	60.9 ± 0.5
*Galium arenarium*	74.4 ± 1.6 **	77.2 ± 0.9 **	63.2 ± 3.6
*Helichrysum stoechas*	70.8 ± 1.6 *	71.4 ± 3.0 *	65.0 ± 4.5
*Limonium vulgare*	71.6 ± 5.0 *	76.5 ± 1.5 **	60.9 ± 2.2
*Matthiola sinuata*	70.9 ± 1.6 *	66.5 ± 7.1 *	61.1 ± 1.8
*Ononis spinosa*	73.6 ± 1.7 *	76.1 ± 1.4 **	62.3 ± 1.9
*Plantago lanceolata*	70.8 ± 4.6 *	70.9 ± 5.2 *	62.3 ± 4.5
*Spergularia marina*	66.3 ± 1.7 *	70.7 ± 2.6 *	58.7 ± 3.6
*Suaeda vera*	72.6 ± 1.9 *	74.7 ± 2.9 *	60.5 ± 1.6
Control	51.5 ± 1.4	62.0 ± 1.2	60.5 ± 1.1

**Table 2:** TNF-α and IL-8 secretions (pg/mL) in supernatant of MDBK and intestinal porcine enterocyte (IPEC-J2) cells exposed to plant extracts and mycotoxin zearalenone (ZEN), deoxynivalenol (DON) and T2. * indicates significantly different means from control cells incubated with mycotoxin alone (*p* < 0.05).

MDBK	TNF-α	IL-8	IPEC-J2	TNF-α	IL-8
Control	102 ± 14	389 ± 55	Control	98 ± 12	343 ± 32
ZEN	95 ± 14	392 ± 44	ZEN	90 ± 10	376 ± 36
*Galium arenarium*	98 ± 12	399 ± 19	*Galium arenarium*	95 ± 11	399 ± 19
*Convolvulus soldanella*	93 ± 3	386 ± 34	*Convolvulus soldanella*	87 ± 5	366 ± 37
*Eryngium campestre*	95 ± 6	391 ± 28	*Eryngium campestre*	97 ± 4	388 ± 23
DON	241 ± 21 *	625 ± 10 *	DON	239 ± 25 *	636 ± 15 *
*Galium arenarium*	85 ± 38	390 ± 26	*Galium arenarium*	88 ± 35	387 ± 27
*Convolvulus soldanella*	83 ± 6	379 ± 8	*Convolvulus soldanella*	87 ± 10	382 ± 11
*Eryngium campestre*	86 ± 9	382 ± 13	*Eryngium campestre*	86 ± 8	388 ± 19
T2	286 ± 13 *	732 ± 25 *	T2	257 ± 22 *	632 ± 28 *
*Galium arenarium*	99 ± 26	376 ± 22	*Galium arenarium*	108 ± 29	401 ± 29
*Convolvulus soldanella*	91 ± 9	429 ± 29	*Convolvulus soldanella*	112 ± 19	398 ± 19
*Eryngium campestre*	105 ± 11	407 ± 20	*Eryngium campestre*	93 ± 10	403 ± 25

**Table 3:** Anti-mycotoxin activity on cell viability of halophyte extracts, expressed in % of IPEC-J2 cell viability. Porcine cells were pre-cultivated in the presence of plant extract (10 μg·mL^−1^), then with each mycotoxin (10 μM). Means ± SD of three replicates are represented, and asterisks indicate significantly different means from control cells incubated with mycotoxin alone (*, *p* < 0.05; **, *p* < 0.01).

Halophytic Species	T2	ZEN	DON
*Convolvulus soldanella*	85.8 ± 1.4 **	71.3 ± 0.5 *	60.1 ± 1.2
*Eryngium campestre*	87.8 ± 0.9 **	75.6 ± 1.2 *	62.6 ± 1.4
*Galium arenarium*	83.4 ± 1.0 **	78.2 ± 1.3 **	62.4 ± 2.1
*Limonium vulgare*	69.6 ± 2.3 *	71.4 ± 3.0 *	64.0 ± 3.7
*Ononis spinosa*	72.9 ± 1.4 *	74.1 ± 1.3 *	60.3 ± 1.4
Control	52.5 ± 1.8	62.9 ± 1.7	60.3 ± 1.4

**Table 4:** NMR spectroscopic data (500 MHz, D_2_O), ^1^H and ^13^C chemical shifts (δ, ppm) as well as ^1^H multiplicities of asperulose identified in MeOH_40_ fraction of *G. arenarium*.(d: doublet, dd: doublet of doublets, m: multiplet, brs: broad singlet).

	Asperuloside (MeOD, 500 MHz)	
Position	^TM^_H_	^TM^_C_, Type
1	5.91 (d)	95.7, CH
3	7.48 (d)	152.7, CH
4	-	107.5, qC
5	3.70 (m)	38.7, CH
6	5.68 (dd)	89.1, CH
7	5.77 (d)	130.9, CH
8	-	144.9, qC
9	3.38	146.2, CH
10	4.65/4.78 (m)	64.3, CH_2_
11	-	176.2, COOH
Ac (CH_3_)	2.1	23.1, CH_3_
Ac (C = O)	-	176.6
1′	4.85 (d)	103.2, CH
2′	3.27 (dd)	75.5, CH
3′	3.51 (m)	79, CH
4′	3.38 (m)	72.1, CH
5′	3.50 (m)	79.1, CH
6′	3.75/3.95 (m)	63, CH_2_

**Table 5:** Effect of mycotoxins (DON, ZEN or T2) and *G. arenarium* extract on trans-epithelial electrical resistance (TEER) values in MDBK cell monolayers. TEER values were normalized as % control. Data represent the mean value ± standard deviation (SD) of three independent experiments in triplicate. * and ^+^ represent mean TEER values significantly different from the negative control and from mycotoxin exposure alone, respectively (*p* < 0.05).

	Control	T2	DON	ZEN
Control	100%	66 ± 10 *	82 ± 15 *	73 ± 6 *
Crude extract	100%	88 ± 5 ^+^	88 ± 4	92 ± 8 ^+^
EtOH fraction	100%	87 ± 6 ^+^	86 ± 8	92 ± 7 ^+^

**Table 6:** Anti-mycotoxin activity on cell viability of *G. arenarium* crude extract and fractions, expressed in % of MDBK cell viability. Bovine cells were pre-cultivated in the presence of plant fraction (10 μg·mL^−1^), then with one of the three mycotoxins at 10 μM. Means ± SD of three replicates are represented, and asterisks indicate significantly different means from the control (*p* < 0.05).

	T2	DON	ZEN
**Crude extract**	74.45 ± 1.6 *	63.23 ± 3.6	77.22 ± 0.9 *
**MeOH_20_**	70.5 ± 2.4 *	99.3 ± 3.2 *	72.5 ± 2.0 *
**MeOH_40_**	69.3 ± 5.9 *	99.2 ± 1.2 *	76.1 ± 0.5 *
**MeOH_60_**	71.6 ± 1.6 *	100.0 ± 3.6 *	77.4 ± 2.3 *
**MeOH_80_**	72.8 ± 2.9 *	100.0 ± 4.8 *	78.4 ± 4.0 *
**MeOH_100_**	76.6 ± 1.2 *	100.0 ± 4.0 *	79.6 ± 1.2 *
**EtOH_100_**	76.1 ± 1.4 *	100.0 ± 2.0 *	82.8 ± 5.1 *
**Control**	51.5 ± 1.4	60.5 ± 1.1	62.0 ± 1.2

**Table 7:** DPPH-scavenging (Inhibitory concentration of 50% (IC_50_), in mg·mL^−1^) and total antioxidant capacity (TAC, in mg AAE·g^−1^ DW) of *G. arenarium* crude extract and fractions. Means ± SD of three replicates are represented, and different letters indicate significantly different means (*p* < 0.05).

	DPPH	TAC
Crude extract	0.072 ± 0.003 ^c^	251.78 ± 4.83 ^c^
MeOH_20_	0.017 ± 0.002 ^a^	304.71 ± 11.46 ^b^
MeOH_40_	0.034 ± 0.001 ^b^	413.89 ± 44.13 ^a^
MeOH_60_	0.014 ± 0.002 ^a^	308.22 ± 2.71 ^b^
MeOH_80_	0.040 ± 0.006 ^b^	315.50 ± 10.13 ^b^
MeOH_100_	0.511 ± 0.013 ^d^	38.38 ± 6.22 ^e^
EtOH_100_	1.000 ± 0.000 ^e^	124.55 ± 5.39 ^d^

**Table 8:** Location and habitat of the twelve studied species.

Scientific Name	Family	Habitat	Locality
*Convolvulus soldanella*	*Convolvulaceae*	Flore dune	Le Conquet (Fr)
*Dianthus gallicus*	*Caryophyllaceae*	Grey dune	Crozon (Fr)
*Eryngium campestre*	*Apiaceae*	Grey dune	Le Conquet (Fr)
*Frankenia laevis*	*Frankeniaceae*	Grey dune	Le Conquet (Fr)
*Galium arenarium*	*Rubiaceae*	Flore dune	Le Conquet (Fr)
*Helichrysum stoechas*	*Asteraceae*	Grey dune	Plouharnel (Fr)
*Limonium vulgare*	*Plumbaginaceae*	Schorre	Fouesnant (Fr)
*Matthiola sinuata*	*Brassicaceae*	Yellow dune	St-Pierre-Quiberon (Fr)
*Ononis spinosa*	*Fabaceae*	Grey dune	Le Conquet (Fr)
*Plantago lanceolata*	*Plantaginaceae*	Grey dune	St-Pierre-Quiberon (Fr)
*Spergularia marina*	*Caryophyllaceae*	Schorre	Le Conquet (Fr)
*Suaeda vera*	*Amaranthaceae*	Schorre	Le Conquet (Fr)

## Data Availability

Data is contained within the article.

## Abbreviations

AAE	Ascorbic Acid Equivalent
DMSO	Dimethyl sulfoxide
DON	Deoxynivalenol
DPPH	2,2-diphenyl-1-picrylhydrazyl
EMEM	Eagle’s Minimum Essential Medium
HSF	Horse fœtal serum
IC50	Inhibitory concentration of 50%
IPEC-J2	Intestinal porcine cell line
MDBK	Bovine kidney cell line
MTS	3-(4,5-dimethylthiazol-2-yl)-5-(3-carboxymethoxyphenyl)-2-(4-sulfophenyl)-2H-tetrazolium; NO, nitric oxide
ZEN	Zearalenone
